# No association between anterior cruciate ligament reconstruction using hamstring autograft and knee flexion strength deficits at 4 months on seated isokinetic testing at 180°/s in adolescents

**DOI:** 10.1002/jeo2.70926

**Published:** 2026-10-06

**Authors:** Maika Bazebi, David Mazy, Zoe David, SiYu Wang, Alex Delisle, Guy Grimard, Marie‐Lyne Nault

**Affiliations:** ^1^ Department of Surgery Université de Montréal Montreal Quebec Canada; ^2^ Azrieli Research Center CHU Sainte‐Justine Montreal Quebec Canada

**Keywords:** adolescents, anterior cruciate ligament reconstruction, hamstring autograft, isokinetic strength, Limb Symmetry Index

## Abstract

**Purpose:**

This study aimed to analyse the effect of using an ipsilateral hamstring (HS) autograft on early postoperative knee strength following anterior cruciate ligament reconstruction (ACLR) in adolescents.

**Methods:**

This retrospective, single‐centre study included patients aged ≤18 years who underwent primary ACLR with HS tendon autografts between January 2022 and January 2025. Demographic and operative data were collected. Isokinetic knee flexion and extension peak torque was assessed bilaterally at 180°/s using a standardized dynamometer protocol, and Limb Symmetry Index (LSI) was calculated preoperatively and at 4 months postoperatively.

**Results:**

Ninety‐three patients met the inclusion criteria (mean age, 15.6 ± 1.3 years). No significant postoperative strength deficits were detected in the operated limb for either flexion (*p* = 0.672) or extension (*p* = 0.316) compared with preoperative values. The contralateral healthy limb demonstrated significant strength gains in both flexion and extension (*p* < 0.001). The LSI decreased significantly postoperatively (*p* < 0.001), with a greater reduction in extension compared with flexion (12% vs. 7%). There were no differences in LSI between female and male patients.

**Conclusions:**

ACLR in adolescents using an ipsilateral HS autograft was not found to be associated with significant postoperative flexion or extension strength deficits on the operated limb at 4 months. The postoperative asymmetry seemed primarily driven by strength gains in the contralateral healthy limb. These findings highlight the importance of not overlooking quadriceps strengthening even when using an HS autograft and support the continued use of this graft choice in adolescents, especially regarding the preservation of flexion strength.

**Level of Evidence:**

Level III, retrospective case‐control study.

**Abbreviations**
ACL: anterior cruciate ligament
ACLR: anterior cruciate ligament reconstruction
HS: hamstring
LSI: Limb Symmetry Index
RTS: return‐to‐sport

## INTRODUCTION

One of the ongoing challenges in rehabilitation after anterior cruciate ligament reconstruction (ACLR) is the persistence of hamstring (HS) strength deficits during the early postoperative phase [6, 27]. Studies report HS strength deficits around 30% at 6 months post‐ACLR [27], and residual deficits of up to 20% are still common at the time of return‐to‐sport (RTS) [1, 31]. Importantly, these deficits may persist long‐term, with structural and functional weakness observed from two to more than 10 years after surgery [1, 3, 33, 42, 43]. In adult patients, these deficits appear to be exacerbated when an ipsilateral HS autograft is used [2, 4, 6, 7, 14, 26, 27, 43]. ACLR with HS autograft can ultimately result in a Grade IV muscle‐tendon injury [6], accompanied by chronic neuromuscular inhibition, proximal migration of the semitendinosus muscle‐tendon junction and selective atrophy of the harvested muscles [6].

The HSs are considered functional agonists of the anterior cruciate ligament (ACL) [6, 28] with a key role as dynamic stabilizers of the knee joint [27]. By generating posterior tibial shear forces, they limit anterior translation of the tibia, reduce excessive ACL loading and contribute to valgus‐varus and rotational stability [6, 34]. Deficits in HS function following ACLR are associated with increased risk of knee osteoarthritis, altered gait mechanics, reduced movement quality and greater risk of re‐injury upon RTS [6]. Addressing these early deficits is therefore essential to optimize functional recovery and minimize long‐term morbidity.

The incidence of ACL injury and ACLR among the paediatric and adolescent populations has increased sharply over the past two decades, at a significantly higher rate than in adults [21, 24]. This rise is largely attributed to greater year‐round sports participation, competitive play and early sport specialization [2, 4, 14, 20, 24, 27, 30, 34]. Another cause for concern is the higher graft failure rate in young patients compared to adults [2, 24, 29, 30, 34], with reported re‐injury rates reaching 40% in some studies [2]. Consequently, a better understanding of the impact of graft selection on knee strength is essential to tailor rehabilitation protocols and a safe progression toward sports readiness in youth athletes. To our knowledge, there have been no studies on early postoperative strength following ACLR with a HS graft in adolescents that also consider preoperative strength status. This gap limits the ability to distinguish true postoperative graft‐related deficits from preexisting injury‐related weakness, despite evidence that preoperative strength substantially influences postoperative recovery trajectories and limb symmetry outcomes [8, 10, 13, 37, 48].

The purpose of this study was to assess whether the use of an ipsilateral HS autograft for ACLR influences postoperative HS and quadriceps strength recovery in adolescents in light of preoperative strength levels. We hypothesized that using this graft would reduce flexion strength during early postoperative isokinetic testing.

## MATERIALS AND METHODS

### Ethics

This study received approval from the institutional ethics review board at CHU Sainte‐Justine, which granted a waiver of consent (No. 2026‐9144).

### Study design

We conducted a single‐centre retrospective cohort analysis that included patients treated between January 2022 and July 2025. Patients were included if they were ≤18 years old, underwent unilateral primary ACLR using an ipsilateral HS autograft and had available preoperative and postoperative isokinetic testing. Patients were excluded if they underwent revision ACLR, contralateral ACLR, multiligament reconstruction or associated osteotomy. We collected demographic data including sex, age, weight and height at the time of surgery, as well as the following surgical data: ACLR surgical technique, concomitant lateral extra‐articular tenodesis (LET) and meniscal surgeries.

### Surgical procedure

Two fellowship‐trained orthopaedic sports surgeons (M.‐L.N. and G.G.) performed all the ACLRs at a single tertiary paediatric centre using an HS tendon autograft. The semitendinosus was harvested first; if the quadrupled graft diameter was <8 mm, the gracilis tendon was also harvested and added to form a six‐strand construct for a final graft diameter of 8–10 mm. All ACLRs were carried out with an inside‐out technique using femoral and tibial adjustable‐loop cortical buttons (Infinity; ConMed). Since 2023, we began adding a concomitant LET to the ACLR, based on evidence supporting its role in reducing graft failure, particularly in young patients with HS autograft and participating in pivoting sports [5, 41]. The LET was performed according to the modified Lemaire technique. A 1 cm × 8 cm strip of the posterior iliotibial band was harvested while preserving its distal attachment to the Gerdy tubercle. After ACL fixation, the strip was passed beneath the lateral collateral ligament and fixed to the femur with a suture anchor (TruShot; ConMed) at 30° of knee flexion in neutral rotation.

### Postoperative rehabilitation

We conducted the postoperative rehabilitation follow‐up according to a standardized institutional ACLR rehabilitation protocol, with supervised physical therapy initiated within 1 week of surgery. Weight‐bearing and knee flexion range of motion (0°–90°) were restricted for the first 6 weeks in cases of meniscal repair.

The early phase (0–6 weeks) emphasized management of acute symptoms, restoration of full knee extension, quadriceps activation and normalized gait. Weeks 6–12 focused on progressive HS and quadriceps strengthening, neuromuscular control training and advanced balance and functional training, with isolated HS strengthening introduced around Weeks 6–8. Isolated HS strengthening consisted of resisted knee flexion exercises performed in the prone position to reduce hip flexion compensation, with the foot positioned beyond the edge of the treatment table to allow antigravity loading. Manual resistance was applied progressively. Light jogging was permitted at approximately 16 weeks when adequate strength and movement quality were demonstrated. Sport‐specific drills were initiated around 8 months once functional testing criteria were achieved. Full RTS was generally permitted at 9–12 months with surgeon clearance.

However, due to the retrospective nature of the study, we could not track individual adherence, attendance and progression throughout the rehabilitation period.

### Outcome measures

We assessed knee flexion and extension peak torques using a Humac NORM isokinetic dynamometer (CSMi). The Humac NORM has shown good‐to‐excellent reliability for knee muscle testing (intraclass correlation coefficient [ICC] = 0.74–0.89) [16]. Testing was performed preoperatively to establish baseline values and again at approximately 4 months postoperatively. This timepoint represents a key clinical milestone, as most return‐to‐running progressions are initiated between 3 and 4 months when specific strength thresholds are achieved [35].

Participants completed a five‐minute warm‐up on a stationary bicycle at low resistance. We tested isokinetic strength in a seated position with the backrest set at 85°. The axis of rotation of the dynamometer was aligned with the lateral femoral epicondyle. The trunk, pelvis and distal thigh were secured using stabilization straps to minimize compensatory movements. The range of motion was set from 0° (full knee extension) to 135° of knee flexion. Gravity correction was applied according to manufacturer guidelines.

We measured concentric knee flexion and extension strength at 180°/s. This velocity aligns with the standardized protocol used in the ACL RTS evaluation pathway at CHU Sainte‐Justine, where slower velocities such as 60°/s are not performed before 5 months due to the increased anterior shear forces generated at low speeds, placing additional stress on the healing graft [47]. According to Humac NORM testing guidelines, although slower velocities are optimal for determining true peak torque, high‐speed testing offers a more functional representation of muscular performance and provides information more closely aligned with the demands of functional activity.

For each limb, we recorded peak torque (Nm) in both flexion and extension, measured strength deficits as a percentage difference between limbs and computed the Limb Symmetry Index (LSI) as (operated/non‐operated limb) × 100. Both peak torque values and LSI scores for flexion and extension were used for analysis. An LSI value of 100% reflects perfect symmetry between both lower limbs. A decrease in LSI indicates increasing asymmetry between the limbs. Isokinetic dynamometry is considered the gold standard for quantifying knee flexion and extension strength and has been validated as a reliable method to assess postoperative muscle recovery and symmetry following ACLR [23, 49].

### Analysis

The first analysis allowed us to compare pre‐ and postoperative isokinetic strength values at 180 °/s on both sides. Values from both the injured and the healthy sides were compared using their absolute values as well as their side‐to‐side differences. Subsequently, we compared both sides to calculate the LSI preoperatively and postoperatively. We used subgroup analyses to evaluate potential differences according to sex and assess the impact of a concomitant meniscal repair on isokinetic testing results.

### Statistical analysis

We performed all statistical analyses using IBM SPSS Statistics (Version 31.0.0.0; IBM Corp.). Normally distributed continuous variables are reported as mean ± standard deviation, while dichotomous variables are expressed as the number and percentage of patients. We assessed the normal distribution of the sample with the Shapiro–Wilk normality test and compared categorical variables between groups with the *χ* ^2^ test and Fisher's exact test (for categories with <5% of the total population). We used a paired *t* test for the pre‐ and postoperative comparison and the independent‐samples *t* test or Mann–Whitney *U* test for continuous variables, depending on the normality test results. Statistical significance was set at *p* < 0.05.

## RESULTS

Ninety‐three patients met the inclusion criteria (Figure 1). Demographic characteristics are presented in Table 1. Pre‐ and postoperative isokinetic strength results are summarized in Table 2. The mean interval between surgery and postoperative isokinetic testing was 4 ± 1.2 months.

**Figure 1:**
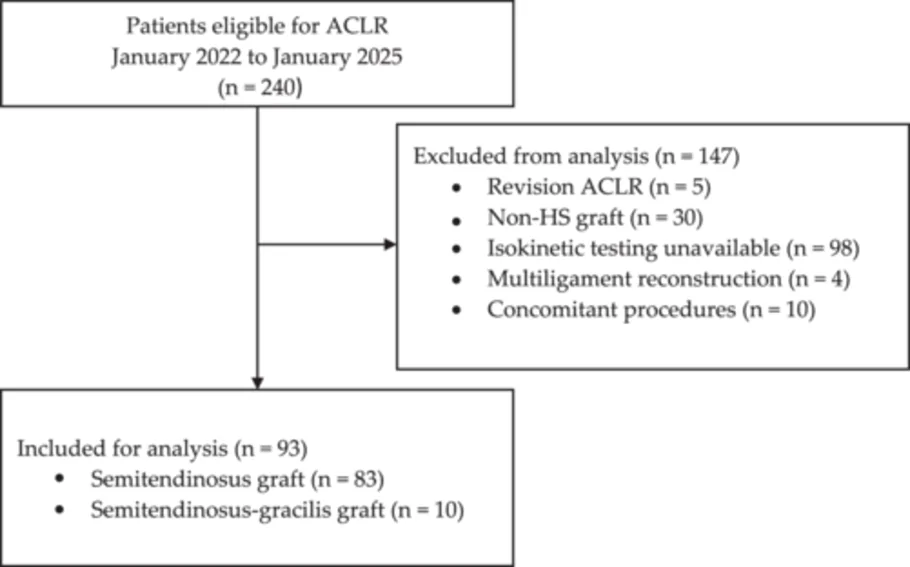
Patient flowchart: Study inclusion and exclusion criteria. ACLR, anterior cruciate ligament reconstruction; HS, hamstring.

**Table 1:** Patient demographics.

*n* = 93	
Age (years), mean ± SD [min; max]	15.6 ± 1.3 [12; 18]
Female, *n* (%)	59 (63%)
Side, right, *n* (%)	47 (51%)
BMI, mean ± SD [min; max]	22.5 ± 4.1 [13.4; 39.8]
ST graft, *n* (%)	83 (89%)
ST + G graft, *n* (%)	10 (11%)
LET, *n* (%)	45 (48%)

Abbreviations: BMI, body mass index; G, gracilis; LET, lateral extra‐articular tenodesis; SD, standard deviation; ST, semitendinosus.

**Table 2:** Pre‐ and postoperative isokinetic test values at 180°/s.

*n *= 93	Pre‐operative	Post‐operative	*p*‐values
Flexion			
Injured side (Nm), mean ± SD	67.6 ± 18.4	68.2 ± 17.8	0.672
Healthy side (Nm), mean ± SD	68.7 ± 16.6	74.4 ± 18.1	**<0.001**
Side‐to‐side difference	1.1 ± 10.8	6.2 ± 9.7	**<0.001**
LSI (%, ± SD)	99 ± 14.2	92.1 ± 13.1	**<0.001**
Extension			
Injured side (Nm), mean ± SD	82.2 ± 22.8	80.5 ± 23.8	0.316
Healthy side (Nm), mean ± SD	90.2 ± 23.4	100.8 ± 26.5	**<0.001**
Side‐to‐side difference	7.9 ± 15.6	20.3 ± 16.1	**<0.001**
LSI (%, ± SD)	92.5 ± 16.4	80.4 ± 15.1	**<0.001**

*Note*: Bold values are statistically significant.

Abbreviations: LSI, Limb Symmetry Index; Nm, Newton‐metres; SD, standard deviation.

### Sex‐based analysis

Sex‐based comparisons are reported in Table 3.

**Table 3:** Sex‐based comparison.

	Female (*n* = 59)	Male (*n* = 34)	*p* value
Age (years), mean ± SD	15.7 ± 1.3	15.4 ± 1.3	0.369
BMI, mean ± SD	22.9 ± 4.1	21.8 ± 4	0.209
Right side, *n* (%)	29 (49%)	18 (53%)	0.725
LET (%)	31 (53%)	14 (41%)	0.291
Preoperative isokinetic tests			
Flexion			
Injured side (Nm), mean ± SD	60.3 ± 10.9	80.4 ± 21.6	**<0.001**
Healthy side (Nm), mean ± SD	61.9 ± 11.2	80.6 ± 17.6	**<0.001**
Side‐to‐side difference	1.6 ± 7.8	0.2 ± 14.8	0.539
LSI (%, ± SD)	98 ± 13.1	100 ± 16.1	0.517
Extension			
Injured side (Nm), mean ± SD	73.9 ± 16	96.5 ± 25.9	**<0.001**
Healthy side (Nm), mean ± SD	81.2 ± 17.1	106 ± 24.8	**<0.001**
Side‐to‐side difference	7.3 ± 13.1	9.5 ± 19.4	0.513
LSI (%, ± SD)	92.5 ± 17	92 ± 15.5	0.886

*Note*: Bold values are statistically significant.

Abbreviations: BMI, body mass index; LET, lateral extra‐articular tenodesis; LSI, Limb Symmetry Index; Nm, Newton‐metres; SD, standard deviation.

### Subgroups analysis

Isokinetic strength values comparing patients with and without meniscal repair are provided in Table 4. The group without meniscal repair included 12 patients who underwent a partial meniscectomy and 42 patients with no meniscal procedure. Meniscal tear characteristics and procedures are summarized in Table 5.

**Table 4:** Subgroup comparison between patients with meniscal suture and without.

	No meniscal repair (*n* = 54)	Meniscal repair (*n* = 39)	*p* value
Age (years), mean ± SD	15.7 ± 1.4	15.5 ± 1.2	0.577
Female, *n* (%)	34 (63%)	25 (64%)	0.910
BMI, mean ± SD	23 ± 4.3	21.7 ± 3.7	0.132
LET (%)	26 (48%)	19 (49%)	0.957
Postoperative isokinetic tests			
Flexion			
Injured side (Nm), mean ± SD	69.9 ± 18.1	65.8 ± 17.3	0.276
Healthy side (Nm), mean ± SD	74.8 ± 17.9	73.7 ± 18.6	0.777
Side‐to‐side difference	4.9 ± 8.7	7.9 ± 10.7	0.141
LSI (%, ± SD)	93.8 ± 12.1	89.9 ± 14.1	0.165
Extension			
Injured side (Nm), mean ± SD	83.3 ± 21.2	76.8 ± 26.8	0.199
Healthy side (Nm), mean ± SD	99.1 ± 24.2	103.1 ± 29.5	0.468
Side‐to‐side difference	15.8 ± 14.8	26.3 ± 16	**0.002**
LSI (%, ± SD)	84.8 ± 13.3	74.3 ± 15.3	**<0.001**

*Note*: Bold values are statistically significant.

Abbreviations: BMI, body mass index; LET, lateral extra‐articular tenodesis; LSI, Limb Symmetry Index; Nm, Newton‐metres; SD, standard deviation.

**Table 5:** Meniscal tear characteristics and surgical management.

Meniscal tear = 51	
Tear location	
Lateral meniscus	32 (63%)
Medial meniscus	8 (16%)
Both	11 (22%)
Type of tear	
Vertical	17 (33%)
Complex	17 (33%)
Bucket‐handle	7 (14%)
Radial	4 (8%)
Parrot‐beak	3 (6%)
Root	2 (4%)
Horizontal	1 (2%)
Procedure	
Sutures (AI or IO)	39 (76%)
Partial meniscectomy	12 (24%)

*Note*: Patient flowchart.

Abbreviations: AI, all‐inside; IO, inside‐out.

We performed a post hoc power analysis to assess the study's ability to detect a significant difference in LSI between preoperative and postoperative measurements. Based on a prespecified effect size of 0.20 and a total sample size of 93 patients, the statistical power was 0.83.

## DISCUSSION

The most important finding of the present study is that ACLR in adolescents using an ipsilateral HS autograft was not found to be significantly associated with postoperative strength deficits in knee flexion or extension at 4 months when compared with their preoperative baseline. Despite the lack of significant postoperative weakness detected in the operated limb, we observed significant limb asymmetry for both muscle groups. Although subtle deficits below the threshold of statistical detection cannot be entirely excluded, the clinical relevance of such small differences remains questionable. Indeed, postoperative strength recovery is usually interpreted using LSI and established RTS thresholds rather than small changes in absolute peak torque.

These findings contradict our hypothesis that HS graft harvest would reduce flexion strength during early postoperative isokinetic testing. Our findings indicated that the observed asymmetry appears to be driven primarily by strength gains in the contralateral limb rather than deficits in the operated limb, as the injured side demonstrated stable and even slightly improved flexion strength postoperatively. The contralateral strength gains observed postoperatively may reflect several independent mechanisms, including neural cross‐education and a familiarization effect related to repeated isokinetic testing. Unilateral resistance training has been shown to induce strength adaptations in the contralateral limb through neural cross‐education mechanisms, although this phenomenon has been more extensively studied in the opposite context (i.e., strength gains in the injured limb resulting from training on the healthy limb) [25]. In addition, Impellizzeri et al. documented a learning effect for HS peak torque measurements, particularly in seated testing positions, that may not replicate functional length‐tension relationships [18]. The use of a familiarization session has been shown to reduce variability in isokinetic outcomes, although practice‐based improvement may still occur across multiple testing sessions [32]. Increased overall rehabilitation exposure may also have contributed to these findings, and these mechanisms likely act in combination during early postoperative recovery.

Interestingly, despite the use of an HS graft, the asymmetry was greater in knee extension than in flexion (12% vs. 7% deficit), falling below commonly acceptable RTS thresholds (≥90%), whereas knee flexion symmetry remained within an acceptable range.

While 4 months postoperative is far too early for RTS, this timepoint is clinically meaningful given that strength LSI at 4 months was shown to be a strong predictor of LSI outcomes at 8 months, a common criterion for RTS readiness [11]. Although Simonsson et al. [40] recently suggested that LSI alone may not fully discriminate athletes who are safe to RTS, their findings, along with others, indicate that an LSI score <90% is associated with increased ACL graft failure, reinjury and reduced odds of safe RTS [9, 15, 22]. When combined with evidence that extension strength deficits have been strongly associated with delayed RTS [45], these findings collectively suggest that early injury‐related quadriceps weakness may represent a more functionally relevant limiting factor during rehabilitation than HS graft harvest‐related impairment.

Notably, knee extension asymmetry was already present preoperatively. Despite postoperative gains in bilateral flexion strength, a reduction in peak knee extension torque was noted; however, none of these variations reached statistical significance. Clinically, these findings may reflect prehabilitation and early rehabilitation strategies prioritizing disproportionate flexion conditioning in anticipation of tendon harvest and may explain the relatively preserved postoperative flexion strength profile. This interpretation is supported by Shaarani et al. [39] and Giesche et al. [13], whose systematic reviews concluded that prehabilitation improves knee function, self‐reported outcomes, neuromuscular performance and RTS rates, with benefits still evident at 12 weeks postoperatively [40]. Riesterer et al. [37] found that preoperative knee extension and flexion strength are strongly correlated with postoperative performance following ACLR, emphasizing the need for comprehensive preoperative screening and targeted strength intervention. Similarly, Scherer et al. [38] reported that both extension and flexion strength are associated with a faster functional recovery after ACLR, highlighting the importance of restoring flexion and extension strength as key objectives of preoperative rehabilitation. Carter et al. [8] supported these findings but noted that there was no consensus regarding the optimal structure of prehabilitation programs.

Our findings align with those of Rakauskas et al. [34], who recently compared 68 adolescents (12–17 years) undergoing ACLR using either HS or quadriceps tendon autograft. They reported no significant postoperative change in knee flexion strength between graft groups and found no differences between the operated and contralateral limbs within the HS cohort at 6 months. Similar to our results, they observed persistent extension weakness on the operated side at 6 and 12 months, even in patients who received an HS graft. However, contrary to our findings, the postoperative deficit in knee extension strength was mainly due to the operated limb relative to the contralateral limb. Notably, their analysis was conducted at 60°/s and did not include preoperative testing, limiting a direct comparison with our results. Comparable results were reported by Weaver et al. [43], who retrospectively reviewed 201 adolescents (12–18 years) undergoing ACLR with HS or quadriceps tendon autograft and found no significant differences in flexion peak torque between graft types at 7–8 months. They also found no significant extension strength asymmetry within the HS group, contrary to our observations. Their cohorts included cases where contralateral semitendinosus harvest was performed when the ipsilateral graft diameter was <8 mm, which may have impacted postoperative strength recovery. Meanwhile, Greenberg et al. [14] reported comparable postoperative knee flexion strength outcomes between graft types in adolescent athletes evaluated at 3, 6 and 12 months at 180°/s, supporting the current findings. Conversely, Bram et al. [4] found lower flexion LSI in the HS group compared with quadriceps graft recipients at 3 months, although this difference was no longer significant by 6 months, suggesting that early asymmetry may normalize over time.

Our study also demonstrated that female adolescents exhibited lower absolute peak torque values than males for both muscle groups, consistent with previous literature reporting sex‐based strength differences in youth athletes [17]. However, despite these absolute strength production discrepancies, preoperative LSI values were comparable between sexes, suggesting preserved proportional symmetry between limbs. These findings may be partially explained by pubertal development and sex‐specific neuromuscular adaptations during growth, which are typically associated with greater increases in strength and power in males [17]. However, such adaptations likely affect both limbs symmetrically and therefore do not alter relative LSIs. Subgroup analysis further revealed greater preoperative asymmetry in knee extension than in knee flexion, supporting the notion that knee extension deficits may be more pronounced than flexion deficits, even prior to surgery. While preoperative flexion symmetry was almost fully restored between limbs (98%–100%), extension LSI remained closer to 92%. Similar observations were reported by Maguire et al. [27], who found comparable LSI values between sexes despite lower absolute strength in females. Weaver et al. [43] also demonstrated lower absolute peak torque values for women, as well as a greater extension asymmetry in both female and male adolescents. Altogether, these data underscore the importance of prioritizing quadriceps strengthening during prehabilitation to address asymmetry before surgery and enhance postoperative recovery trajectories.

Subgroup analysis comparing patients who underwent meniscal repair with those without repair found no significant association with postoperative peak torque value deficits or deficits in LSI scores regarding flexion strength within the context of our study design, parameters and testing conditions. However, a significant difference was observed in extension LSI, favouring patients without repair. Overall, extension LSI values were approximately 10% lower than flexion values. This finding suggests that postoperative restrictions commonly implemented following meniscal suturing, such as limited weight‐bearing and restricted knee flexion for up to 6 weeks, may disproportionately impair quadriceps recovery while having less effect on flexion strength. These results further highlight the importance of targeted quadriceps strengthening in rehabilitation protocols for patients undergoing concomitant meniscal repair. Our results partially align with Weaver et al. [44], who reported no effect of meniscal tear type and treatment during ACLR on quadriceps peak torque or symmetry at 6–9 months post‐ACLR in adolescents. However, similarly to our study, they demonstrated greater asymmetry in flexion than in extension, especially after meniscal repair. Conversely, Redler et al. [36] found that adolescents with meniscal repair had greater flexion strength deficits at 6 months than those without repair. Differences in rehabilitation protocols and testing timepoints may explain these discrepancies. Taken together, these findings highlight the need for future studies to evaluate the influence of meniscal tear characteristics and postoperative restrictions on strength recovery trajectories in adolescents to optimize rehabilitation protocols.

Our findings suggest that adolescents may benefit from rehabilitation protocols that are more focused on restoring extension strength, beginning in the preoperative phase and continuing throughout early postoperative recovery. Given the pronounced extension asymmetry observed in extension compared with flexion, particularly in patients undergoing meniscal repair, protocols may require targeted quadriceps loading strategies to mitigate early strength loss and support safer RTS progression.

This study is not without limitations. First, its retrospective design is associated with potential confounding factors, namely variability in prehabilitation protocols and rehabilitation adherence, which may have influenced strength outcomes. As such, causal inference cannot be established. Furthermore, rehabilitation adherence was not formally tracked, which may influence results and introduce a potential bias. Second, strength assessment was limited to a single postoperative timepoint, preventing our evaluation of longer‐term recovery trajectories or RTS readiness. In addition, strength testing was performed at a single angular velocity (180°/s), in accordance with the institutional protocol designed to protect the healing graft during early rehabilitation. Although it is common to use higher angular velocities (i.e., 180°/s) in postoperative assessment, they may yield lower peak torque values compared with lower testing velocities such as 60°/s, traditionally used to assess maximal strength [19]. Therefore, this approach may provide a more conservative estimate of maximal torque production, which should be considered when interpreting the absence of significant postoperative strength deficits. Furthermore, using LSI to assess strength represents an additional limitation. Indeed, LSI values are inherently influenced by contralateral limb performance and may not fully reflect isolated recovery of the injured limb, potentially leading to over‐ or underestimation of the strength recovery in the injured limb [46]. Recent literature has questioned the use of LSI as a standalone marker of functional recovery and RTS readiness, emphasizing that symmetry‐based metrics do not account for absolute strength levels and should be interpreted within a broader clinical context. For this reason, both absolute peak torque values and LSI were reported in the present study to provide a more comprehensive assessment of postoperative strength recovery. Finally, strength testing was performed in a seated position, which may not fully capture maximal active knee flexion range and could potentially mask deficits occurring outside the tested range of motion [12]. Third, all procedures were performed at a single tertiary paediatric centre by two surgeons using standardized protocols, and a follow‐up was conducted by experienced ACL rehabilitation physical therapists, which may limit generalizability to other clinical settings. Additionally, the study did not include a comparison cohort treated with an alternative graft, precluding direct evaluation of graft‐specific effects. No subgroup analyses were performed for patients with partial meniscectomy or no meniscal surgery due to the small size of these subgroups. Similarly, no subgroup analyses were conducted to assess the potential influence of concomitant LET procedures or graft composition due to the limited number of patients with combined semitendinosus and gracilis grafts. These factors may have influenced the observed outcomes. Future research should include prospective randomized trials with standardized and monitored prehabilitation and rehabilitation protocols and adequately powered comparisons between graft types and adjunctive procedures to better define strength recovery in adolescents after ACLR.

## CONCLUSION

In this cohort of paediatric patients undergoing ACLR with an HS ipsilateral autograft, isokinetic testing revealed a greater strength asymmetry in extension than in flexion at 4 months postoperative, without statistically significant peak torque deficits being detected relative to preoperative baseline values. A postoperative protocol limiting weightbearing and knee flexion after concomitant meniscal repair was not found to be associated with additional flexion deficits, but extension strength was again the most affected parameter. While these findings should be interpreted within the context of the early postoperative timepoint at 4 months and the specific seated isokinetic testing protocol at 180°/s and the absence of a comparator graft, they suggest that HS autograft harvest may not be the primary driver of early postoperative strength deficits, underscoring the need for more quadriceps‐targeted prehabilitation and rehabilitation strategies to optimize recovery.

## AUTHOR CONTRIBUTIONS

Maika Bazebi and David Mazy drafted the manuscript. Maika Bazebi, David Mazy and Marie‐Lyne Nault were responsible for the research design. Maika Bazebi and Zoe David, SiYu Wang and Alex Delisle were responsible for data acquisition. Maika Bazebi, David Mazy, Guy Grimard and Marie‐Lyne Nault analysed and interpreted the data. All authors reviewed and approved the final manuscript.

## FUNDING INFORMATION

The authors have no funding to report.

## CONFLICT OF INTEREST STATEMENT

Marie‐Lyne Nault: The institution (CHU Sainte‐Justine) received departmental funding for research and educational purposes from OrthoPaediatrics and Smith & Nephew. The remaining authors declare no conflicts of interest.

## ETHICS STATEMENT

This study was approved by CHU Sainte‐Justine project no. 2026‐9144.

## Data Availability

Data are available from the corresponding author upon reasonable request.
